# Effect of information encounter on concerns over healthy eating– mediated through body comparison and moderated by body mass index or body satisfaction

**DOI:** 10.1186/s12889-023-15069-0

**Published:** 2023-02-06

**Authors:** Piper Liping Liu, Angela Chang, Matthew Tingchi Liu, Jizhou Francis Ye, Wen Jiao, Harris Song Ao, Weixing Hu, Kaibin Xu, Xinshu Zhao

**Affiliations:** 1 Department of Communication / Institute of Collaborative Research, University of Macau, Macau, China; 2 Department of Communication, University of Macau, Macau, China; 3 Department of Management and Marketing, University of Macau, Macau, China; 4 Department of Communication / Center for Research in Greater Bay Area, University of Macau, Macau, China; 5 Department of Communication / Institute of Collaborative Research / Center for Research in Greater Bay Area, University of Macau, Macau, China; 6 Faculty of Social Sciences / Center for Research in Greater Bay Area, University of Macau, Macau, China; 7 School of Journalism and Communication, Wuhan University, Wuhan, China; 8 Department of Communication, Faculty of Social Sciences, University of Macau, Room 2051, E21B, Humanities and Social Sciences Building, Avenida da Universidade, Taipa, Macau China

**Keywords:** Information encounter, Information scanning, Healthy eating concerns, Body comparison, BMI, Body satisfaction, Average percent effect (*a*_*p*_), Percentage coefficient (*b*_*p*_), Percent contribution (*c*_*p*_)

## Abstract

**Background:**

Understanding factors that influence healthy or unhealthy eating can inform intervention strategies. This study ascertained whether and how unintentional exposure to food and nutrition information influenced healthy eating concerns. The study tested body comparison, body satisfaction, and body mass index as three mechanisms that potentially link food information encounter, commonly known as information scanning, to healthy eating concerns.

**Methods:**

A sample of 440 online participants (mean age = 29.15 years) was used to investigate: (1) how unintentional exposure to food and nutrition information, i.e., information encounter (IE), affects healthy eating concerns (HEC); (2) how the effect of IE on HEC is mediated by body comparison (BC); (3) how the paths of the mediation model are moderated by body satisfaction (BS) or body mass index (BMI).

**Results:**

The findings show a positive and sizable total effect of IE on HEC – a whole-scale increase in information encounter is associated with a substantial increase in healthy eating concerns by 15 percentage points (*b*_*p*_ = 0.150). BC is found to mediate the effect of IE on HEC in an all-positive complementary mediation. Both the indirect and the direct-and-remainder paths show sizable effects. The mediated path contributes about 20% of the total effect between IE and HEC (*c*_*p*_ = 20%), while the direct-and-remainder path contributes the rest (*c*_*p*_ = 80%). BS was found to moderate the relationship between IE and BC, the first leg of the mediation. The moderation effect is large – the effect of IE on BC is much smaller on the highly and the moderately satisfied than on the lowly satisfied (slope differential *b*_*p*_ = -.60). BMI was found to moderate the direct-and-remainder effect of IE on HEC, controlling BC. That is, the effect of IE on HEC, after filtering out the mediated effect through BC, is much larger for those with high or low BMI than those with healthy BMI (slope differential *b*_*p*_ = .32).

**Conclusions:**

Exposure, even if unintentional, to food and nutrition information is an important predictor of HEC. BC, BS, and BMI are important factors that help to explain the process through which information affects behaviors.

## Background

Food choices and eating patterns have changed dramatically over the decades around the world. Diets have shifted toward increased consumption of ultra-processed foods that are energy-dense, low in nutrients, and high in fat [1, 2]. Unhealthy dietary patterns have been found associated with obesity [3], depression [4], metabolic changes [5], and an increased risk of chronic diseases such as colorectal cancer [6] and adenomas [7]. Promoting healthy eating is thus of vital importance and urgency.

*Information* *seeking versus encounter*. The media powered by information technology are powerful channels for messages about food, nutrition, and eating [8, 9]. Exposure to media information may be intentional or accidental, i.e., active or passive [10–12]. Active information seeking is vital, as it reduces uncertainty and reinforces decisions made [13]. In general, individuals who actively seek health-related information, such as guidance for health and nutritional food [14, 15] or risks associated with emerging infectious diseases [8], are typically in the middle of practicing desired behaviour for health promotion. For instance, Beaudoin and Hong found that seeking health information from traditional media, e.g., newspapers and television could entice healthy behaviour such as fruit and vegetable consumption [14].

Not all information acquisition is active. Unintentional and accidental encounters with information are common in daily life and work [12]. For example, people may be passively exposed to food and nutrition information in a regular TV program, or they may obtain the information accidentally while browsing social media [16]. Such passive encounter with information has been labelled “information scanning” following the pioneering work of Kosicki and McLeod in the 1980s, which were later adopted by researchers across disciplines, including especially health researchers in the era of the internet [8–10, 12, 14–18].

Seeking and scanning are not the only concepts for active and passive communication. Attention and exposure, for example, were from the era of television [19–22]. Information enquiry and encounter might be alternative labels for information seeking and scanning, as discussed below.

*Information scan or encounter.* Daily connotation of “scanning” sound more active than the academic definition in the “seeking versus scanning” literature discussed above. The popular thesaurus.com [23] lists the top synonyms of “scan” as “browse, check, examine, flash, flip through, leaf through, look through, scour, search, skim, thumb through,” all of which sound more active than passive. As “seeking” and “scanning” sound more like synonyms than antonyms, some scholars define “information seeking” as one mode of “environmental scanning [24].” Accordingly, this study uses “information encounter” for the traditionally labelled “information scanning.” For alliteration, “information enquiry versus encounter” may be an alternative pair that provide closer matches between connotations and concepts than “seeking versus scanning”.

This study investigates whether and how *information encounter* (IE), defined as unintentional, accidental, and passive exposure to information, influences one’s concerns about eating and nutrition, i.e., choosing healthier food.

IE is a more prevalent and more effective than intentional seeking [10, 17]. However, little is known about the moderation and mediation process through which IE impacts eating behaviour. To fill this gap, the present study aims to (1) investigate the influence of food and nutrition IE on healthy eating concerns (HEC); (2) assess how IE is associated with body comparison (BC), and how BC influences HEC; (3) examine the mediating role of BC and moderating role of body mass index (BMI) and body satisfaction (BS) on HEC.

### Hypotheses and research questions

#### Hypotheses

IE is defined as “the information acquisition that occurs within routine patents of exposure to mediated and interpersonal sources that can be recalled with a minimal prompt” [12]. A burgeoning body of research reports a positive association between health information scanning, i.e., encountering, and healthy behaviours. Shim and colleagues found that cancer information encounter was associated with increased knowledge about cancer and lifestyle choices preventing cancer [25]. Similarly, Bigsby and Hovick's study also lend support to the positive influence of health information encounter on health decision-making such as exercise and fruit and vegetable intake [26]. Thus food and nutrition IE may increase one’s concerns about food and eating, which entail our first hypothesis:

- H1: Food and nutrition IE is positively associated with HEC.

There may be more than a bivariate association between IE and HEC. Sociological and psychological literature emphasizes the cognitive mechanisms through which media exposure affects behaviors [27–29]. Social Comparison Theory [30–32] explicates that people evaluate themselves (e.g., abilities, attractiveness, and intelligence) in relation to those of others, which may significantly impact self-image, behavior, and psychological well-being [33–35]. There are two types of SC, i.e., downward comparison of oneself to the less fortunate [30], and upward comparison of oneself to the superior [30, 31].

Upward comparisons encourage self-promotion when discrepancies are perceived [27]. Bessenoff reported that BC mediated the association between media exposure and self-esteem because BC could arouse people’s concerns over weight and appearance [27]. Likewise, Yun and Silk found that the more people paid attention to information that might evoke upward SC, the more likely they were to show stronger intentions to exercise and maintain a healthy diet [36]. Thus, the second hypothesis related to a positive pathway from food and nutrition IE to upward BC, and to HEC, is proposed:

- H2: Food and nutrition IE has a positive indirect effect on HEC through BC. That is, the IE is positively correlated with BC, which is positively correlated with HEC.

Hypotheses 1 and 2, together, imply a complementary mediation in which the direct and indirect paths are both positive [37]. It has been shown mathematically that the total-effect test for complementary mediation always passes the *p* < α threshold [38], which means that Hypotheses 1 and 2 also imply Hypotheses 3 below.

- H3: IE is positively associated with HEC without controlling for BC.

As stated, H3 implies a positive total effect of IE on HEC, which may be tested by whether the simple regression coefficient of the effect of IE on HEC passes the *p* < 0.05 threshold.

On top of the mediation model of IE, BC and HEC implied in the above three hypotheses, this study also investigates the moderation effects of two potential moderators, BS and BMI. BMI evaluates body weight relative to height to determine overall mass. BMI below 18.5 is considered underweight, above 23.9 is overweight including obesity, while between the two numbers is considered a healthy BMI [39].

BS refers to the complex psychological construct of individuals’ feelings and evaluations about the weight and shape of his/her body [40, 41]. BS differs between individuals. Those unsatisfied with their body tend to gain or lose weight. Low BS is associated with changes in eating behavior, such as higher levels of dieting awareness, frequent dieting, and even unhealthy weight control behaviors (e.g., vomiting, laxatives, diet pills) [42–44]. Those with lower BS or unhealthy BMI may be more susceptible to BC facilitated by media messages and were more concerned about food and eating [42, 45].

**Fig. 1:**
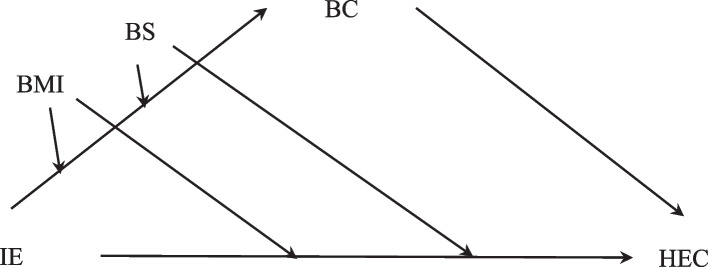
Hypothesized model of moderated mediation. Note: IE = Information encounter; BC = Body comparison; HEC = Healthy eating concerns; BS = Body satisfaction; BMI = Body mass index

This study thus constructed a moderated-mediation model to examine whether the direct influence of food and nutrition IE on HEC and the mediation effect of BC were moderated by BS and BMI (see Fig. 1). Four hypotheses were proposed:

- H4: The indirect effect of food and nutrition IE on HEC through BC is contingent on individuals’ BS such that the effect is stronger for those who have lower levels of BS.
- H5: BMI moderates the indirect effect of food and nutrition IE on HEC through BC such that the effect is stronger for those with unhealthy weight.
- H6: BS moderates the direct relation between food and nutrition IE and HEC, with the relation being stronger for individuals who have lower levels of BS.
- H7: BMI moderates the direct relation between food and nutrition IE and HEC, with the relation being stronger for individuals with unhealthy weight.

#### Research questions

Traditional hypothesis testing often has been criticized for its binary nature, which limits its outcomes to yes–no or pass-fail, thereby restricting the scope of scientific inquiry [46–53]. For mediation and moderation analyses, one recommended remedy is to consider establishing hypothesized mediation or moderation only as a necessary first step and focus more on understanding model types and analyzing effect sizes [37, 38, 54]. In response are the following research questions:

- Q1: What type of mediation or non-mediation best characterizes the effect of IE on HEC through BC?

Hypotheses 1 and 2 together imply a complementary mediation, which is defined as a model for which the direct and indirect paths point in the same direction and both pass the statistical threshold *p* < α, where α is traditionally pre-set at 0.05, 0.01, 0.001, etc. [37, 38]. H1 and H2 both predict positive effects. If one or both hypotheses fail, it would still be worth knowing what type of mediation or non-mediation best describes the data.

- Q2: What type of moderation emerges, if one or more of the hypothesized moderation effects (H4-H7) is supported?

Mediation and moderation are models of causal dissection. They both dissect, or partition, the total effect of the independent variable on the dependent variable, aka X–Y total effect [55–58]. The logic behind the mediation typology also applies to moderation. This study attempts to apply a developing typology for moderation to real data [37, 38, 54, 59].

- Q3: How large are the effects of various parts of the moderated mediation models?

While following the tradition of reporting regression coefficients (*b*), this study will also adopt statisticians’ and methodologists’ recommendations to place variables on 0–1 percentage scales (*p*_*s*_) [60–62]. With all variables on 0–1 scales, regression coefficients (*b*) become *percentage coefficients* (*b*_*p*_), which allows normalized interpretation and comparisons [37, 38, 63, 64]. To answer Q3, we will compare each effect with the 0–1 percentage scale and compare various component effects with each other, e.g., direct (*d*) vs. indirect (a*b), first-leg (*a*) vs. second-leg (*b*), and each component effect with the total effect (*c*).

## Method

Data for this study are a part of a 38-country survey, Corona Cooking Survey (CCS). The missions of the survey were to investigate the relationship between the media, communication, and people’s food-related behaviours, such as buying, cooking, and eating habits, during the COVID-19 pandemic, and to identify possible shifts and differences in the behaviours [65]. Specifically, the questionnaire includes a wide array of questions on media use, body image, eating concerns and behaviours, food buying and cooking behaviours, health perception, health status, and demographics. The survey is under the umbrella of a larger multi-year project, Food, Media and Society (FOOMS), at the University of Antwerp (UAntwerp) in Belgium.

### Procedure and samples

Respondents were recruited via a convenience sample for adults aged 18 and above. This represented our best option for identifying and securing participants during the lockdown amid the COVID-19 pandemic. Even though convenience sampling runs a high risk that our sample will not perfectly represent the population, the method still has its uses, in particular, when we researchers need to conduct a nationwide study quickly and on a shoestring budget. Additionally, we followed the project protocol and practices pursuing consistency among all researchers involved in all countries [65].

A web-based questionnaire with snowball sampling was employed for Chinese participants who live in Mainland China. Other recruitment tools were also employed to increase sample diversity. First, multiple channels such as email circulation were employed. Second, social media were designed and shared by the research team on sites (e.g., Wechat and Weibo) in both private and public online groups. In addition, to motivate potential participants, a lucky draw was arranged for fully completed questionnaires, with a maximum payout of MOP2,000 for 20 participants. A written informed consent was obtained from every subject before data collection. Participation was voluntary, confidential, and anonymous and had a median duration of 35 min. Eventually, 476 Chinese adults from mainland China, Taiwan, Hong Kong, and Macao were invited to complete the CCS questionnaire (response rate = 92.4%). Only respondents aged 20 and above were involved for the data analysis (*n* = 440).

The data collection took place November–December 2020 for all countries after approval by the Research Ethics Committee of the Social Sciences and Humanities, UAntwerp. Qualtrics software was employed to produce a uniform online questionnaire, while the questions were translated and back-translated several times into local languages to minimize the possible differences in meanings.

### Measurement

Food and nutrition information encounter (IE) was measured by a question taken from prior research [11]. Participants were first informed of the media sources that they may be exposed to food and nutrition-related messages include cookbooks, food-related social media, cooking shows, websites, blogs about food, etc., while advertisements were excluded. They were then required to indicate “to what extent you had come across media messages about food and nutrition coincidentally or spontaneously”. Responses were scored on a five-point Likert scale, ranging from 1 = never to 5 = always (M = 2.87, SD = 0.70). Although single-item measure for IE, aka information scanning, is considered acceptable per prior publication [11], the inter-measure reliability and thereby the validity may be improved significantly by multi-item measures, which future studies may need to consider.

Body satisfaction (BS) was measured by four items adopted and adapted from previous studies [44]. Respondents were asked to evaluate their satisfaction with four aspects of their body: (1) overall appearance; (2) overall body; (3) weight; and (4) muscle tone/size. Response options ranged from extremely dissatisfied (= 1) to extremely satisfied (= 7). A composite variable was computed by averaging the four items (M = 3.74, SD = 1.03, Cronbach’s alpha = 0.84).

Body comparison (BC) was measured using three items drawn from previous research [45], on a five-point Likert scale continuum from 1 (*strongly disagree*) to 5 (*strongly agree*). Respondents were asked to rate the extent to which media messages concerning food allow them to: (1) watch people’s bodies; (2) compare their bodies to those of others; and (3) fantasize about an ideal body. The three items were averaged to create an index (M = 3.09, SD = 0.57, Cronbach’s alpha = 0.70), with higher values representing higher levels of body comparison.

Healthy eating concerns (HEC) were measured with three items adapted and modified based on previous research [66, 67]. Respondents were required to indicate if they were concerned about whether the food they ate on a typical day (1) would keep them healthy; (2) was high in vitamins/minerals; and (3) was nutritious. A five-point Likert scale was used (1 = never, 5 = always), and the answers were averaged to create the measure of HEC (M = 3.25, SD = 0.58). The inter-measure reliability (α = 0.69) barely missed the conventional threshold of 0.7, threatening its validity; caution is needed when interpreting related outcomes.

BMI (Body mass index) was calculated with weight in kilograms divided by height in meters squared (height: M = 1.64, SD = 0.87; weight: M = 56.88, SD = 11.27; BMI: M = 21.27, SD = 3.12). Furthermore, BMI was categorized into two groups: (1) healthy weight group with a range between 18.5 and 23.9 (77.0%); and (2) unhealthy weight group with the BMI below 18.5 (17.3%) or above 23.9 (5.7%).

Demographics include age, gender (1 = female, 2 = male, 3 = other), education (1 = under a high school diploma or none, 2 = high school diploma or equivalent, 3 = bachelor’s degree or equivalent, 4 = master’s degree or equivalent, and 5 = doctorate), were queried.

### Analytical tools and approach

SPSS version 22 was used for the data analysis. First, to make variables comparable, all focal variables were converted into a common measurement scale of 0 to 1. For instance, we can subtract 1 from a five-point rating to adjust the scale to start at 0, and then divide it by 4 to compress the scale (see Table 1 for details). Second, to test the moderation-mediation model, Hayes' SPSS PROCESS (model 8) was used [68]. In this model, IE and HEC served as the independent and dependent variables, respectively, with BC as the mediator, and BMI and BS as two moderators. Bootstrapping technique (*N* = 5,000) and 95% confidence intervals (CI) were used to statistically test the moderation and mediation effects. The effect should be considered tenable if the 95% CI does not include zero. This study also experiments to heed the advice of statisticians, journal editors, and academic societies to use *p* values appropriately and to place more emphasis on effect size measures [69–72].

**Table 1:** Descriptive statistics of variables (*N* = 440)

		Original Scale				0 -1 Percentage Scale			
#	Variables	Min	Max	Mean	Sd	Min	Max	Mean	Sd
1	Age	20	79	29.15	11.07	0	1	0.155	0.188
2	Gender (1 = being male)	0	1	0.407	0.492	0	1	0.407	0.492
3	Education	1	5	2.67	0.85	0	1	0.418	0.212
4	IE	1	5	2.87	0.70	0	1	0.468	0.176
5	BC	1	5	3.09	0.57	0	1	0.521	0.143
6	BS	1	7	3.74	1.03	0	1	0.456	0.171
7	HEC	1	5	3.25	0.578	0	1	0.563	0.144

*IE* Information encounter, *BC* Body comparison, *HEC* Healthy eating concerns, *BS* Body satisfaction, *BMI* Body mass index

## Results

### Hypothesis testing

Hypothesis 1 predicted that food and nutrition IE will be positively associated with HEC. As depicted in Table 2, the direct IE-HEC path past the statistical threshold (*b*_*p*_ = 0.12, *p* = 0.002).

**Table 2:** Effect of IE on HE mediated through BC and moderated by BS or BMI^a^

	Effect (*b*_*p*_)^2^	SE	95%CI	*p*
IE → BC (a)	.17	.04	[.090, .243]	< .001
(BC → HEC)/IE (b)	.20	.05	[.111, .295]	< .001
(IE → HEC)/BC (d)	.12	.04	[.042, .195]	.002
IE → BC → HEC (a*b)	.03	.02	[.008, .081]	.005
IE → HEC (c)	.15	.04	[.054, .253]	< .001
	BS	Effect (*b*_*p*_)^b^	Boot SE	95%CI
Conditional indirect effect*	M – 1SD	.05	.02	[.017, .102]
	M	.03	.02	[.005, .069]
	M + 1SD	.01	.02	[-.021, .049]
	BMI	Effect (*b*_*p*_)^b^	Boot SE	95%CI
Conditional direct effect*	Healthy	.04	.05	[-.058, .130]
	Unhealthy	.36	.06	[.239, .488]

^a^* IE* Information encounter, *BC* Body comparison, *HEC* Healthy eating concerns, *BS* Body satisfaction, *BMI* Body mass index

^b^* b*_*p*_ stands for *percentage coefficient*, which is regression coefficient (*b*) when independent and dependent variables are both on 0–1 percentage scales (*p*_*s*_)

Hypothesis 2 posited that food and nutrition IE would have an indirect effect on HEC, mediated by BC. Results in Table 2 show that food and nutrition IE was positively associated with BC (*b*_*p*_ = 0.17, *p* < 0.001), and BC was positively associated with HEC (*b*_*p*_ = 0.20, *p* < 0.001). The indirect effect was statistically acknowledged (95% CI: [0.008, 0.081], p = 0.005), supported by the bootstrapping results.

Hypothesis 3 predicted a positive total effect of IE on HEC, which is supported by a positive association between IE and HEC without controlling for the mediator, BC (*b*_*p*_ = 0.15, *p* < 0.001).

**Fig. 2:**
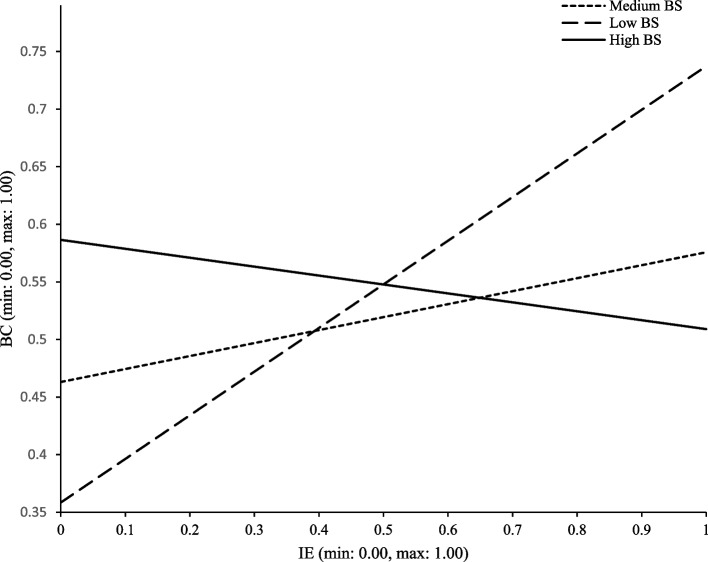
Moderating effect of BC on IE-BC path (first leg of indirect path). Note: IE = Information encounter; BC = Body comparison; BS = Body satisfaction

Hypothesis 4 and 5 postulated the moderation effects of BS and BMI in the indirect association between food and nutrition IE and HEC, mediated by BC. As shown in, Table 2 for individuals with low (mean – 1SD) and medium (mean) BS, the indirect effects were statistically acknowledged (low BS: 95% CI: [0.017, 0.102]; medium BS: 95% CI: [0.005, 0.069]). For descriptive purposes, Fig. 2 plotted food and nutrition IE on BC, separately for low, medium, and high levels of BS (1 SD below the mean, mean, and 1 SD above the mean, respectively). Simple slope tests showed that for individuals with low and medium BS, food and nutrition IE was positively associated with BC. However, for people with high S, the IE-BC relation was statistically inconclusive. In addition, no moderation effect of BMI in the indirect association between food and nutrition IE and HEC was found.

**Fig. 3:**
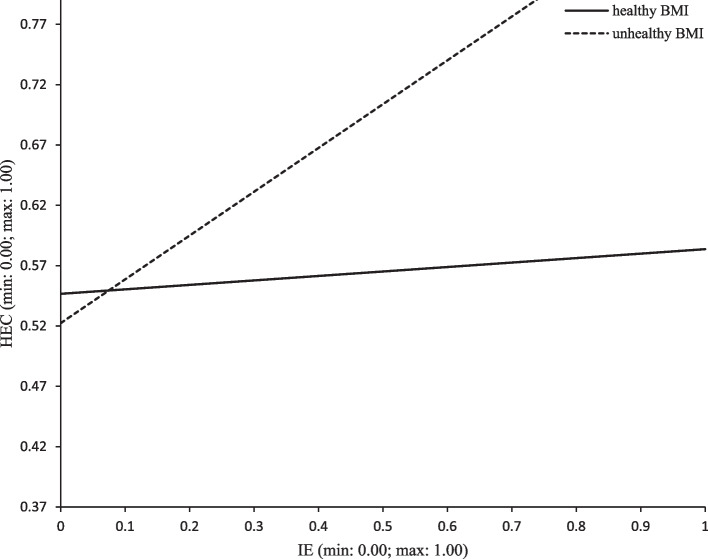
Moderating effect of BMI on IE-HEC path (direct path). Note: IE = Information encounter; HEC = Healthy eating concerns; BMI = Body mass index

Hypothesis 6 and 7 predicted the moderation effects of BS and BMI in the direct association between food and nutrition IE and HEC. As depicted in Table 2 and illustrated in Fig. 3, for people with unhealthy BMI, food and nutrition IE was positively related to HEC (95% CI: [0.239, 0.488]), whereas no moderation effect of BS in the direct relation between food and nutrition IE and HEC was found.

### Addressing research questions

**Fig. 4:**
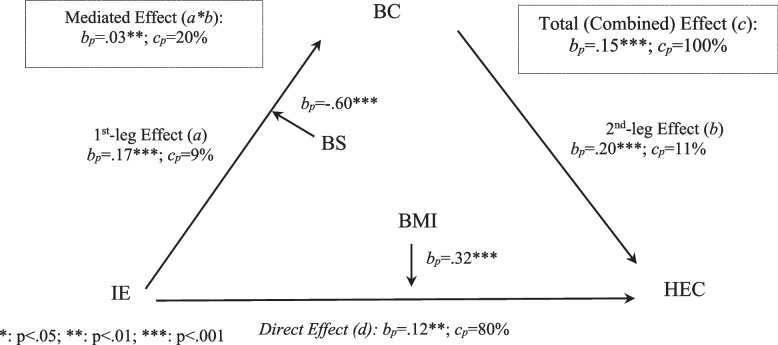
The effect of IE on HEC mediated through BC and moderated by BS or BMI. *: *p* < .05; **: *p* < .01; ***: *p* < .001. Note: IE = Information encounter; BC = Body comparison; HEC = Healthy eating concerns; BS = Body satisfaction; BMI = Body mass index

Research Questions 1 and 2 inquire about the types of mediation and moderation models. The mediation model reported above (Fig. 4) is *complementary*, given that the mediated (a*b) and the direct (*d*) paths both pass the predetermined statistical threshold *p* < 0.05 and they point at the same direction (positive) [37, 38]. The finding is consistent with a corollary implied in H1 and H2, as mentioned earlier.

The two models of moderation (Figs. 2 and 3) are both *uni-group*, given that in each model one group (i.e., the *low* BC and *medium BC* group in Fig. 2 and the *unhealthy* group in Fig. 3) shows an effect passing the *p* < 0.05 test while the other group(s) (i.e., the *high BC* groups in Fig. 2 and the *healthy* group in Fig. 3) fails the test. Defined as a moderation model in which one group shows an effect while the other group fails, uni-group moderation parallels indirect-only mediation [37, 38, 54].

With all variables on 0–1 percentage scales, a regression coefficient (*b*) indicates a percentage change in the dependent variable associated with a whole-scale increase from conceptual minimum (0) to conceptual maximum (1) in the independent variable. This particular regression coefficient is referred to as the percentage coefficient (*b*_*p*_) [37, 38, 64]. A *b*_*p*_ = 0.17 (*p* < 0.001) for the relation between IE and BC (Table 2 and Fig. 4), for example, indicates that a whole-scale rise in IE from 0 to 1 is linked with an increased BC by 0.17 points, also on a 0–1 scale. With this understanding, we interpret and compare the percentage coefficients (*b*_*p*_) in Table 2 and Fig. 4 to address Research Question 3, which requires us to assess and analyze effect sizes.

The total effect of IE on HEC, *b*_*p*_ = 0.15 (*p* < 0.001), estimates the final product, or outcome, of the process under analysis. It is important, therefore, to understand the size of this effect before dissecting the process that produced it. While 0.15 appears sizable against the 0–1 scale, it appears more impressive against the observed mean dependent variable, HEC, which is 0.563 (Table 1, Line 7). Thus, a maximum increase in IE appears related to an increase in HEC by over 26.64% (0.15/0.563), with the observed average HEC as the baseline. One may also assume mean independent variable (IS = 0.468, Line 4) and calculate the *average percent effect* (*a*_*p*_): *a*_*p*_ = *b*_*p*_ $\overline{X }$*/*$\overline{Y }$= 0.15 × 0.468/0.563 = 0.125, suggesting that an average amount of IE is linked with a 12.5% increase in HEC from the observed average. Average percent effect (*a*_*p*_) is thus a comprehensive measure of an aspect of the effect, taking into consideration three key pieces of information, efficiency (*b*_*p*_), mean dependent variable ($\stackrel{-}{X)}$ and mean independent variable ($\stackrel{-}{Y)}$.

The mediation model dissects this total effect into two paths, indirect effect (*b*_*p*_ = 0.03, *p* = 0.005) and direct effect (*b*_*p*_ = 0.12, *p* = 0.002). Of the two paths, the direct effect is much larger than, (i.e., four times as much) its indirect complement (*b*_*p*_ = 0.12 vs *b*_*p*_ = 0.03). The *p* values are closer to each other (*p* = 0.002 vs *p* = 0.005), adding another example that *p* values are not good approximators of effect sizes. Of the mediated path, the second leg appears slightly more efficient (*b*_*p*_ = 0.20, *p* < 0.001) than the first (*b*_*p*_ = 0.17, *p* < 0.001).

The percentage coefficients can be used to calculate *percent contribution (c*_*p*_*),* defined as the contribution to the total effect of X on Y by each component effect, expressed as the percent figure (%), of a mediation model. It so happens that the component paths, *a, b, c, d,* and hence a*b, of this model are all positive, which simplifies the calculation and the interpretation. Tables 3 and 4 below detail the *c*_*p*_ calculation assuming all paths are zero or above.

**Table 3:** Percent contributions to mediation total effect

${c}_{p(c)}=\frac{{b}_{p\left(c\right)}}{{b}_{p\left(c\right)}}$	1
${c}_{p\left(ab\right)}=\frac{{b}_{p\left(ab\right)}}{{b}_{p\left(c\right)}}$	2
${c}_{p\left(d\right)}=\frac{{b}_{p\left(d\right)}}{{b}_{p\left(d\right)}}$	3
${c}_{p\left(a\right)}=\frac{{b}_{p\left(a\right)}}{{b}_{p\left(a\right)}+{b}_{p\left(b\right)}}{c}_{p\left(ab\right)}$	4
${c}_{p\left(b\right)}=\frac{{b}_{p\left(b\right)}}{{b}_{p\left(a\right)}+{b}_{p\left(b\right)}}{c}_{p\left(ab\right)}$	5

All paths (*a*, *b*, *c*, *d* and a*b) are assumed non-negative, i.e., 0 ≤ *b*_*p*_ ≤ 1, which is appropriate for the data of this study

**Table 4:** Concepts, sings, and definitions of Table 1

	Concept	Definition
1	*b*_*p(c)*_	Percentage coefficient (*b*_*p*_) of the total effect, aka *c* path
2	*b*_*p(ab)*_	Percentage coefficient (*b*_*p*_) of the mediated effect, aka a*b path
3	*b*_*p(d)*_	Percentage coefficient (*b*_*p*_) of the direct effect, aka *d* path
4	*b*_*p(a)*_	Percentage coefficient (*b*_*p*_) of the first leg of the mediated effect, aka *a* path
5	*b*_*p(b)*_	Percentage coefficient (*b*_*p*_) of the second leg of the mediated effect, aka *b* path
6	*c*_*p(c)*_	Percent contribution of the total effect, aka *c* path, to the total effect *c*
7	*c*_*p(ab)*_	Percent contribution of the mediated effect, aka a*b path, to the total effect *c*
8	*c*_*p(d)*_	Percent contribution of the direct effect, aka *d* path, to the total effect *c*
9	*c*_*p(a)*_	Percent contribution of the first leg of the mediated effect, aka *a* path, to the total effect *c*
10	*c*_*p(b)*_	Percent contribution of the second leg of the mediated effect, aka *b* path, to the total effect *c*

The *c*_*p*_ figures show that the direct path accounted for 80% of the total effect of IE on HE, while the mediated path accounted for the other 20% (Fig. 4). The so-called “direct path” (*d*) encompasses the effects of all possible mediators unidentified in the mediation model of IS-BC-HEC in addition to the possibly “true” direct effect, thus is more accurately “direct-and-remainder path” [37, 55, 73, 74]. The 20% due to a single mediator (BC) appears significant indeed, not just “statistically” significant.

Of the 20% contribution by the mediated path, less than half (*c*_*p*_ = 9%) is attributed to the first leg and more than half (*c*_*p*_ = 11%) to the second leg (Fig. 4). Note that *c*_*p*_ for the mediated and the direct-and-remainder paths add up to 100%, and the *c*_*p*_ for the first and the second legs add up to the *c*_*p*_ for the mediated path.

The two *b*_*p*_ coefficients for the moderation effects (*b*_*p*_ = -0.60 for BS and *b*_*p*_ = 0.32 for BMI) appear large, whether against 0–1 scale or against *b*_*p*_ figures for the mediation model, which range from *b*_*p*_ = 0.03 for a*b to *b*_*p*_ = 0.20 for *b* (Fig. 4). The sizes of the moderation effects are also shown visually in Figs. 2 and 3 by the differences between slopes.

## Summary and discussion

Guided by social comarison theory, this study shows direct and indirect effects of food-and-nutrition IE on HEC mediated by BC. BS moderated the first leg of the indirect path and BMI moderated the direct-and-remainder path.

### Theoretical contributions

The hypothesis that BC mediates the relationship between IE and HEC was supported. Unintentional exposure to food and nutrition information may motivate people to compare themselves with an ideal body, thereby increasing HEC. At the same time, the direct-and-remainder path was positive and statistically acknowledged, producing a model of complementary mediation [37, 38].

Three possible explanations appear available. First, information encounter may help to increase the exposure to and recall of food and nutrition information, which in turn inspires HEC thanks to the benefits, risks, and the advocates and advice by food and nutrition professionals for HEC embedded in such messages. Second, given the trend on digital media where people post or view images or advice about fitness, healthy eating, and nutrition‐related behaviors [75], encounters with food and nutrition information may reinforce descriptive or subjective norms. Information encounters may encourage the normative belief that a healthy body is expected, and most people engage in healthy eating activities to maintain healthy body weight. As such, a predisposition to engage in BC could be an underlying mechanism (herein referred to as a mediator) influencing one’s concerns about healthy eating. Third, repeated exposure to food and nutrition information could make the reasons for healthy eating more cognitively accessible [17]. Especially for behaviors that require high levels of commitment (e.g., regular exercise and healthy eating), IE is likely to serve as a good reminder for individuals to recall the long-term benefits of healthy food consumption, elevate healthy eating concerns, and motivate persistent healthy eating behaviors.

The moderating effects of BS and BMI deserves notice. BS moderated the first leg of the mediated path. IS-BC effect is stronger for those with lower BS, that is, IE showed a stronger effect on body comparison for those who are less satisfied with their own bodies – percentage coefficient *b*_*p*_ = 0.03 for the respondents with mean BS, *b*_*p*_ = 0.05 for those with lower BS (1 SD below mean), and *b*_*p*_ = 0.01 for those with higher BS (1 SD above mean). Prior studies reported that, regardless of BMI status, body satisfaction or dissatisfaction tended to be cultivated by one’s social environment, including media messages [45, 76]. The mass media are powerful conveyors of sociocultural ideals that play a critical role in the development of body dissatisfaction. Those who were less satisfied with their own bodies might be more vulnerable to body comparisons, hence more susceptible to the influence of information related to food and nutrition.

BMI moderated the direct path. Controlling BC, the IS-HEC effect was weaker for those with medically recommended BMI (*b*_*p*_ = 0.04, Table 2), and stronger for those with higher or lower BMI (*b*_*p*_ = 0.36). The difference between the two slopes, *b*_*p*_ = 0.32, was statistically acknowledged at *p* < 0.001 (Figs. 2 and 3). As being overweight and underweight entail health risks [77–79], people with unhealthy BMI may have been more concerned with their health, leading to a stronger effect of food-and-nutrition information on HEC.

### Practical implications

Several practical implications may be derived. First, that IE was positively associated with HEC suggests that information acquired unintentionally can also impact health-related decision-making concerns and behaviors. Health information campaigns may consider using diverse media platforms that complement each other to encourage IE, and maximize both intended and coincidental encounters with food and nutrition information [26].

The so-called “direct path” encompasses all routes and mechanisms, direct and indirect, identified and unidentified, that are unaccounted for by the mediators explicitly identified in a model of mediation [37, 38]. In that sense, the term “direct path” could be a misnomer. With this understood, a mediator contributing single-handedly 10% to the X–Y total effect should be considered significant – substantively significant, but not just “statistically significant,” the latter of which often misleads the uninitiated.

Against this background, the 20% (*c*_*p*_ = 0.02) of the IS-HEC total effect contributed by the indirect path is significant indeed. It suggests that body comparison constitutes a crucial mechanism through which food and nutrition information promotes HEC, or at least the perceptions and concerns about it. People may be motivated by food and nutrition information that depicts fitness and health, evoking fantasies of “ideal” body shapes, which may impact concerns and perceptions about eating behaviors. What’s ideal for some, however, may be distorted, unhealthy, or even harmful from a medical perspective, leading to weight and eating disorders. As such, information intervention programs that promote healthy eating, such as nutrition education and dietary guidelines, may need to take into consideration the mediating role of body comparison, and be sensitive to possibly adding pressure about weight and physical appearance that may exacerbate the psychological, behavioral, and physical disorders.

At the same time, the 80% (*c*_*p*_ = 0.08) contributed by all other factors under the sometimes misleading banner of “direct effect” should not be overlooked. It suggests that body comparison is not the only route through which IE exerts its impact on HEC. The combined effects of the other routes and factors are about four times of the effect through BC. While the research community strives to identify and ascertain the other routes of the process, health promoters and educators may also keep an eye on the other potential mediators emerging from their practice and experience.

Third, the moderation effects suggest that different strategies may be designed or executed for different population segments. For one example, IE was more strongly and positively associated with HEC of those with unhealthy BMI (Fig. 3), strongly suggesting that the information campaign should focus more on the people with unhealthy BMI. On the other hand, IE was more strongly and positively associated with BC for those with low or medium body satisfaction (Fig. 2); considering that irrational BC might cause maladaptive outcomes such as eating disorders and depression [80, 81], information campaigns may exercise caution when encouraging IE by these two groups.

We hasten to note the study’s limitations. First, the cross-sectional design weakens the confidence in causal inference. Replications and verifications are called for, especially with experimental and causal designs. Second, the single-item measure of IE increases the chances of measurement error and reduces reliability. Replications with multiple-item measures are called for. Third, online sampling restricted to selected institutions in the Chinese mainland entails sampling bias. Older adults might be underrepresented, for one example. Probability sampling of all Chinese regions, including Taiwan, Hong Kong, and Macau, would be ideal.

## Conclusion

This study extended the understanding about the effect of food-and-nutrition IE on HEC by formulating and analyzing a moderated mediation model. The evidence shows that IE was a vital antecedent of HEC, while BC served as an important mediator between IE and HEC. BS moderated the positive effect of IE on BC, while BMI moderated the direct effect of IE on HEC. The knowledge may inform the design and execution of culturally-conscious interventions for healthy eating.

## Data Availability

The datasets generated and/or analysed during the current study are not publicly available due constraints from the host of the CCS research group but are available from the co-author A. Chang on reasonable request.

## Abbreviations

HEC: Healthy eating concerns
BC: Body comparison
BS: Body satisfaction
BMI: Body mass index
IE: Food-and-nutrition information encounter
CCS: Corona Cooking Survey
FOOMS: Food, Media and Society
CI: Confidence intervals
